# Thoughts on a very acidic symbiosome

**DOI:** 10.3389/fmicb.2015.00816

**Published:** 2015-08-07

**Authors:** Bor L. Tang

**Affiliations:** NUS Graduate School for Integrative Sciences and Engineering, National University of SingaporeSingapore, Singapore

**Keywords:** algae, coral, symbiosome, symbiosis, zooxanthellae

The symbiosis between Scleractinian corals and dinoflagellate algae of the *Symbiodinium* genus drove the formation of coral reefs since the mid-Triassic. Ecologically important as this may be, surprisingly little is known about the cellular and molecular aspects of the coral-algae symbiosome, composed of a host-derived membrane encasing one or more algae cells further surrounded by membranes of the latter's own making. Bidirectional exchange of metabolic materials across the symbiosome membrane is expected to be complex and intense. However, other than morphological insights offered by histological and microscopic analysis, the components and their activities that might sustain these processes are only beginning to be investigated in recent years via genomic and transcriptomic approaches (Davy et al., 2012; Meyer and Weis, 2012).

An important physiological characteristic of the symbiosome is its luminal pH. This value would, intuitively, be important for functional sustenance of the symbiotic relationship. However, like many other physiological parameters, it is difficult to determine in living symbionts. In this regard, a recent report by Tresguerres and colleagues (Barott et al., 2015) indicating that the symbiosome space has a very low pH of ~4, is intriguing. If supported by other approaches, such a low luminal pH would not only highlight several previously unheralded aspects of the symbiotic physiology, but may have important implications for aspects of symbiosome biogenesis and breakdown.

## Why should the symbiosome lumen be so acidic?

An earlier study has indicated that membrane-bound compartment surrounding *Symbiodiniums* of the sea anemone *Anemonia viridis* is not markedly acidic (Rands et al., 1993). There are, however, reasons to believe that the symbiosome would be slightly acidic compared to the coral cytoplasm, and there is some prior evidence that it is. Venn and colleagues had previously probed the intracellular pH (pHi) of endodermal cell preparations from the coral *S. pistillata* and *A. viridis* using the fluorescent pH sensor dye SNARF-1 (or rather a cell permeant acetoxymethyl ester acetate form of SNARF-1) (Venn et al., 2009). For *S. pistillata* harboring algae symbionts, the authors reported a pHi of 7.13 in the dark, which increased to 7.41 under illuminated conditions. Interestingly, the immediate cytoplasmic area surrounding the algae cell has a greenish fluorescent hue against the yellowish background, indicating that these areas have a distinctly lower pH than the rest of the host cytoplasm. The calibration curves only allowed the authors to set an upper limit of pH < 6 for the symbiosome. In the latter report, the dye lysosensor green D-189, which accumulates in acidic compartments, was used (Barott et al., 2015). The dye effectively labeled the symbiosome lumen, and despite autofluorescnce interference from the algae chlorophyll, a basal value of ~pH 4 could be discerned.

It should be noted that the pH value of 4 is rather low for an intracellular compartment. The pH of mammalian lysosomes is between 4.5 and 5.0 (Mindell, 2012), and that of vacuoles in higher plants between 5.0 and 5.5 (Taiz, 1992). Dinoflagellates are not known to be acidophiles, and such a low pH in the symbiosome lumen would definitely stress the symbiont. The question therefore is how this low pH could be useful? The Authors' results provided a hint, as bafilomycin, which dissipates the pH gradient between coral cytoplasm and symbiosome lumen, lowered oxygen production (a proxy for photosynthesis rate) by the algae. The low luminal pH could thus be part of a carbon concentrating mechanism imposed by the host to boost photosynthetic productivity of the symbiont. Within the symbiosome lumen, the low pH would likely promote the reverse reaction catalyzed by the algae carbonic anhydrase, converting HCO${}_{3}^{-}$ to CO_2_. Although the algae cells are surrounded by a membrane complex generated by them, CO_2_ could presumably cross these by diffusion. The Authors have also proposed that the sharp pH gradient between the coral cytoplasm and the symbiotic lumen could facilitate bidirectional transport of key metabolites by proton gradient-coupled co-transport. These are interesting and valid interpretations, but more concrete evidence awaits further work.

## When and how is the symbiosome acidified?

Another interesting point made by the paper pertains to the identity of the proton pump involved in achieving the low pH. The Authors immune-localized a coral vacuolar (V)-type proton ATPase to the symbiosome membrane, and showed that the cytoplasm-symbiosome pH gradient could be partially dissipated by bafilomycin, a rather specific inhibitor of the vacuolar-type proton pumps. There may well be other mechanisms at work to sustain such a steep pH gradient. One such possible candidate is the evolutionarily conserve P-type proton ATPase which are specifically expressed in symbiotic but not free-living zooxanthellae (Bertucci et al., 2010). When first discovered, this proton pump was proposed to be an initiative by the symbiont to acidify the symbiosome lumen for bicarbonate dehydration. However, in light of the current finding it may well be acting to deacidify the algae cytoplasm against the pH gradient.

That a V-type proton pump serves to acidify the symbiosome is interesting because this type of proton pump is the same as ones in endolysosomal and vacuolar compartments in animals and plants (Marshansky et al., 2014). It is generally agreed that symbionts that are acquired from the water column enter the coral host via a phagocytic process. The establishment of symbiosis, phagosomal maturation should however be arrested at some point, for otherwise the internalized algae would be simply digested in mature endolysosomes. How the phagosomal maturation gets arrested, and subsequently converted to a symbiosome, is an intriguing but understudied process. Recently completed genome sequencing of Cnidarians, including that of the coral *Acropora digitifera*, revealed that these have a surprisingly high degree of genomic complexity that match that of vertebrates (Chapman et al., 2010; Shinzato et al., 2011). Two important set of cellular components that are probably functionally conserved are those that regulate membrane trafficking and innate immunity. These may connect symbiosome pH to its mode of biogenesis and demise.

In a sense, establishment of algal symbiosis is analogous to a foreign invasion, but with the invaders avoiding destruction by the host. There are many different examples of how intracellular bacterial pathogens subvert cellular membrane trafficking mechanisms (Stein et al., 2012), particularly those that could eventually establish an intracellular vacuolar niche for subsequent replication. Early studies in symbiotic dinoflagellate endocytic uptake have indicated that these escape host digestion by somehow preventing the fusion of lysosomes with the vacuole in which they reside (Fitt and Trench, 1983). That the dinoflagellate symbiont manipulates host response to promote survival has been proposed and discussed (Schwarz, 2008; Weis et al., 2008; Davy et al., 2012), and Schwarz (2008) has suggested that symbiont bleaching may be a manifestation of a “release from symbiont-mediated suppression of host cells.” A mechanistic example of how phagosomal maturation is arrested involves the binding of early endosomal Rabs (such as Rab4 and Rab5) but prevent the binding of late endosomal Rab7 to the phagosome (Stein et al., 2012). Interestingly, this is what is observed for the endosomal Rabs of the sea anemone, *Aipstasia pulchella*. ApRab4 and ApRab5 are associated with newly formed zooxanthelllae containing phagosomes (Chen et al., 2004; Hong et al., 2009), while ApRab7 is excluded (Chen et al., 2003). In several cases in bacterial infected mammalian cells, these late endosome Rab excluded bacterial phagosomes do not become acidified, and they do not receive lysosomal enzymes. There are also those that are acidified to some extent but do not receive lysosomal enzymes as lysosomal fusion is prevented. The symbiosome may be analogous to the latter type, and the host coral could specifically target V-ATPase to the forming symbiosome membrane but lysosomal fusion is somehow avoided. Whether this is indeed the case for corals requires further molecular identification and the development of genetic and immunological tools for the analysis of their endomembrane system.

Another structure that may share the same acidic feature as the symbiosome is the auto-lysosomes, which is the fusion product of autophagosomes with lysosomes. Degradation of symbionts by a process of autophagy, termed symbiophagy, has been shown as a mechanism for coral bleaching (Downs et al., 2009). In vertebrates, autophagy is a key mechanism mediating innate immunity (Münz, 2014), and symbiophagy may be analogous to the process of xenophagy, or the removal of invading pathogens. It is unclear at the moment if symbiophagy is only triggered under conditions of stress, or if it also occurs in a more controlled manner at some basal level for homeostatic regulation of symbiont number. One could imagine that being in comparable acidity with lysosomes and autolysosomes, inhabitants of the symbiosome lumen are already in a rather precarious environment. Should the host decide to devour the endosymbionts, there appears to be little need to even induce the formation of autophagosomes *de novo*. In other words, overly simplified as this may sound, all the host needs to do is target lysosomal proteins to a pH compatible and digestion-ready compartment. It has been suggested that the dinoflagellate symbiont has ways of inducing immune tolerance of itself by the host's innate immune system (Detournay et al., 2012). Another interesting notion in this regard has been expounded by Bosch (2013), who proposed that the components of the innate immune system have evolved in early metazoans to control the resident beneficial microbial symbionts, not invasive pathogens.

## Who's controlling whom?

The symbiotic relationship between corals and algae is usually perceived as mutualistic. However, several authors have drawn comparisons with other host-microbe interactions and suggest that the algal zooxanthellae as being fundamentally parasitic in nature (Weis et al., 2008; Malcolm and April, 2012). Upon internalization into a potential coral host, these are able to prevent phagosomal maturation and persist within the intracellular habitat, with an ecological manifestation of mutualism. One way the algae symbiont is able to do that is to mimic the output of a digesting phagosome with photosynthate and other products [in the case of *A. digitifera*, perhaps cysteine (Shinzato et al., 2011)]. From the discussion of Tresguerres and colleagues (Barott et al., 2015), it would appear that the Authors are expounding the opposite, namely that the coral host is in full control of its algal endosymbiont. By acidifying the symbiosome, in addition to actively enhancing the photosynthetic productivity of the symbiotic algae, it would also constrain their cell division (Davy et al., 2012). The algae in this case appears to be an unwilling party entrapped in a intracellular compartment by its predatory captor, where survival could only be ensured if it continues to serve the host's nutritional needs in a generally nutrient poor tropical shallow water environment.

There are clearly aspects of coral-algae symbiosis that are not satisfactorily explained by either of the modes above. One such aspect is the apparently high host-symbiont species fidelity (Santos et al., 2004), which fits neither a parasitic nature of zooxanthellae, nor a predatory mode of endosymbiont acquisition by the corals. Another aspect is the prominent but poorly understood process of coral calcification (which permitted reef formation), which is apparently stimulated by the presence of algal symbionts in hermatypic corals. Calcification of the host involves the calicodermis cells which harbor no symbionts, and it is obvious from existence of non-reef forming corals that calcification is not mandatory for symbiosis, and that it occurs only for specific pairings of coral and algae symbiont. Although this appears to be symbiont driven, there is currently a lack of molecular links between the symbionts and host calcification. There is ample evidence for the notion that calcification is light-enhanced (Moya et al., 2006; Tentori and Allemand, 2006). It has also been shown that light scattering by coral skeletons enhances light absorption by symbiotic algae (Enríquez et al., 2005), but it is otherwise difficult to ascertain how calcification may enhance symbiont fitness or survival. On the other hand, an enhanced calcification rate could be largely due to elevated respiration rate of the host, symbiont, or both. It has been suggested that respiration rate elevations, which could result from increase temperature and nutricnt-enlarged symbiont numbers, may paradoxically precede the subsequent loss of reef building capacity (Wooldridge, 2014).

## Epilogue

As discussed above, evidence for a highly acidic coral-algae symbiosome may have wide-ranging implications both in terms of the symbiotic relationship itself, as well as the genesis and demise of the symbiosome (and its tenants). One awaits future confirmation of the low pH values by other approaches, such as the use of compartment targeting pH sensitive fluorophores (pHluorin) when genetic manipulation tools for coral and algae become available. Transgenesis of *Hydra* by embryo microinjection, for example, is now fairly established (Juliano et al., 2014). The study of Cnidarians with its surprisingly complex genome and symbionts is entering an exciting era, as molecular and cellular analyses beckon.

### Conflict of interest statement

The author declares that the research was conducted in the absence of any commercial or financial relationships that could be construed as a potential conflict of interest.

## References

[B1] Barott K. L. Venn A. A. Perez S. O. Tambutté S. Tresguerres M. (2015). Coral host cells acidify symbiotic algal microenvironment to promote photosynthesis. Proc. Natl. Acad. Sci. U.S.A. 112, 607–612. 10.1073/pnas.1413483112 25548188 PMC4299235

[B2] Bertucci A. Tambutté E. Tambutté S. Allemand D. Zoccola D. (2010). Symbiosis-dependent gene expression in coral-dinoflagellate association: cloning and characterization of a P-type H+-ATPase gene. Proc. Biol. Sci. 277, 87–95. 10.1098/rspb.2009.1266 19793745 PMC2842621

[B3] Bosch T. C. (2013). Cnidarian-microbe interactions and the origin of innate immunity in metazoans. Annu. Rev. Microbiol. 67, 499–518. 10.1146/annurev-micro-092412-155626 23808329

[B4] Chapman J. A. Kirkness E. F. Simakov O. Hampson S. E. Mitros T. Weinmaier T.. (2010). The dynamic genome of Hydra. Nature 464, 592–596. 10.1038/nature08830 20228792 PMC4479502

[B5] Chen M. C. Cheng Y. M. Hong M. C. Fang L. S. (2004). Molecular cloning of Rab5 (ApRab5) in *Aiptasia pulchella* and its retention in phagosomes harboring live zooxanthellae. Biochem. Biophys. Res. Commun. 324, 1024–1033. 10.1016/j.bbrc.2004.09.151 15485657

[B6] Chen M. C. Cheng Y. M. Sung P. J. Kuo C. E. Fang L. S. (2003). Molecular identification of Rab7 (ApRab7) in *Aiptasia pulchella* and its exclusion from phagosomes harboring zooxanthellae. Biochem. Biophys. Res. Commun. 308, 586–595. 10.1016/S0006-291X(03)01428-1 12914791

[B7] Davy S. K. Allemand D. Weis V. M. (2012). Cell biology of cnidarian-dinoflagellate symbiosis. Microbiol. Mol. Biol. Rev. 76, 229–261. 10.1128/MMBR.05014-11 22688813 PMC3372257

[B8] Detournay O. Schnitzler C. E. Poole A. Weis V. M. (2012). Regulation of cnidarian-dinoflagellate mutualisms: evidence that activation of a host TGFβ innate immune pathway promotes tolerance of the symbiont. Dev. Comp. Immunol. 38, 525–537. 10.1016/j.dci.2012.08.008 23010490

[B9] Downs C. A. Kramarsky-Winter E. Martinez J. Kushmaro A. Woodley C. M. Loya Y.. (2009). Symbiophagy as a cellular mechanism for coral bleaching. Autophagy 5, 211–216. 10.4161/auto.5.2.7405 19066451

[B10] Enríquez S. Méndez E. R. Iglesias-Prieto R. (2005). Multiple scattering on coral skeletons enhances light absorption by symbiotic algae. Limnol. Oceanogr. 50, 1025–1032. 10.4319/lo.2005.50.4.102520856275

[B11] Fitt W. K. Trench R. K. (1983). Endocytosis of the symbiotic dinoflagellate *Symbiodinium microadriaticum* Freudenthal by endodermal cells of the scyphistomae of *Cassiopeia xamachana* and resistance of the algae to host digestion. J. Cell Sci. 64, 195–212. 6141174 10.1242/jcs.64.1.195

[B12] Hong M. C. Huang Y. S. Song P. C. Lin W. W. Fang L. S. Chen M. C. (2009). Cloning and characterization of ApRab4, a recycling Rab protein of *Aiptasia pulchella*, and its implication in the symbiosome biogenesis. Mar. Biotechnol. 11, 771–785. 10.1007/s10126-009-9193-2 19459008

[B13] Juliano C. E. Lin H. Steele R. E. (2014). Generation of transgenic Hydra by embryo microinjection. J. Vis. Exp. e51888. 10.3791/51888 25285460 PMC4828061

[B14] Malcolm H. April H. (2012). The magnesium inhibition and arrested phagosome hypotheses: new perspectives on the evolution and ecology of Symbiodinium symbioses. Biol. Rev. Camb. Philos. Soc. 87, 804–821. 10.1111/j.1469-185X.2012.00223.x 22404964

[B15] Marshansky V. Rubinstein J. L. Grüber G. (2014). Eukaryotic V-ATPase: novel structural findings and functional insights. Biochim. Biophys. Acta 1837, 857–879. 10.1016/j.bbabio.2014.01.018 24508215

[B16] Meyer E. Weis V. M. (2012). Study of cnidarian-algal symbiosis in the “omics” age. Biol. Bull. 223, 44–65. 10.2307/41758993 22983032

[B17] Mindell J. A. (2012). Lysosomal acidification mechanisms. Annu. Rev. Physiol. 74, 69–86. 10.1146/annurev-physiol-012110-142317 22335796

[B18] Moya A. Tambutté S. Tambutté E. Zoccola D. Caminiti N. Allemand D. (2006). Study of calcification during a daily cycle of the coral *Stylophora pistillata*: implications for ‘light-enhanced calcification’. J. Exp. Biol. 209, 3413–3419. 10.1242/jeb.02382 16916976

[B19] Münz C. (2014). Regulation of innate immunity by the molecular machinery of macroautophagy. Cell. Microbiol. 16, 1627–1636. 10.1111/cmi.12358 25244600

[B20] Rands M. Loughman B. Douglas A. (1993). The symbiotic interface in an alga-invertebrate symbiosis. Proc. R. Soc. B 253, 161–165. 10.1098/rspb.1993.0097

[B21] Santos S. R. Shearer T. L. Hannes A. R. Coffroth M. A. (2004). Fine-scale diversity and specificity in the most prevalent lineage of symbiotic dinoflagellates (Symbiodinium, Dinophyceae) of the Caribbean. Mol. Ecol. 13, 459–469. 10.1046/j.1365-294X.2003.02058.x 14717900

[B22] Schwarz J. (2008). Understanding the intracellular niche in Cnidarian-Symbiodinium symbioses: parasites lead the way. Vie Milieu 58, 141–151.

[B23] Shinzato C. Shoguchi E. Kawashima T. Hamada M. Hisata K. Tanaka M.. (2011). Using the *Acropora digitifera* genome to understand coral responses to environmental change. Nature 476, 320–323. 10.1038/nature10249 21785439

[B24] Stein M. P. Müller M. P. Wandinger-Ness A. (2012). Bacterial pathogens commandeer Rab GTPases to establish intracellular niches. Traffic 13, 1565–1588. 10.1111/tra.12000 22901006 PMC3530015

[B25] Taiz L. (1992). The plant vacuole. J. Exp. Biol. 172, 113–122. 9874729 10.1242/jeb.172.1.113

[B26] Tentori E. Allemand D. (2006). Light-enhanced calcification and dark decalcification in isolates of the soft coral Cladiella sp. during tissue recovery. Biol. Bull. 211, 193–202. 10.2307/4134593 17062878

[B27] Venn A. A. Tambutté E. Lotto S. Zoccola D. Allemand D. Tambutté S. (2009). Imaging intracellular pH in a reef coral and symbiotic anemone. Proc. Natl. Acad. Sci. U.S.A. 106, 16574–16579. 10.1073/pnas.0902894106 19720994 PMC2757848

[B28] Weis V. M. Davy S. K. Hoegh-Guldberg O. Rodriguez-Lanetty M. Pringle J. R. (2008). Cell biology in model systems as the key to understanding corals. Trends Ecol. Evol. 23, 369–376. 10.1016/j.tree.2008.03.004 18501991

[B29] Wooldridge S. A. (2014). Assessing coral health and resilience in a warming ocean: why looks can be deceptive. Bioessays 36, 1041–1049. 10.1002/bies.201400074 25303686

